# Accelerated Method for Simulating the Solidification Microstructure of Continuous Casting Billets on GPUs

**DOI:** 10.3390/ma18091955

**Published:** 2025-04-25

**Authors:** Jingjing Wang, Xiaoyu Liu, Yuxin Li, Ruina Mao

**Affiliations:** 1 School of Information Engineering, Shandong Youth University of Political Science, Jinan 250103, China; wangjingjing@sdyu.edu.cn (J.W.); 220026@sdyu.edu.cn (Y.L.); mrn@sdyu.edu.cn (R.M.); 2 School of Information Science and Engineering, Northeastern University, Shenyang 110819, China

**Keywords:** GPU-accelerated method, parallel CA-DCSA algorithm, microstructure simulation, dendrite morphology, continuous casting, speedup

## Abstract

Microstructure simulations of continuous casting billets are vital for understanding solidification mechanisms and optimizing process parameters. However, the commonly used CA (Cellular Automaton) model is limited by grid anisotropy, which affects the accuracy of dendrite morphology simulations. While the DCSA (Decentered Square Algorithm) reduces anisotropy, its high computational cost due to the use of fine grids and dynamic liquid/solid interface tracking hinders large-scale applications. To address this, we propose a high-performance CA-DCSA method on GPUs (Graphic Processing Units). The CA-DCSA algorithm is first refactored and implemented on a CPU–GPU heterogeneous architecture for efficient acceleration. Subsequently, key optimizations, including memory access management and warp divergence reduction, are proposed to enhance GPU utilization. Finally, simulated results are validated through industrial experiments, with relative errors of 2.5% (equiaxed crystal ratio) and 2.3% (average secondary dendrite arm spacing) in 65# steel, and 2.1% and 0.7% in 60# steel. The maximum temperature difference in 65# steel is 1.8 °C. Compared to the serial implementation, the GPU-accelerated method achieves a 1430× higher speed using two GPUs. This work has provided a powerful tool for detailed microstructure observation and process parameter optimization in continuous casting billets.

## 1. Introduction

The mechanical properties of castings heavily depend on the microstructure of billets, especially the ECR (Equiaxed Crystal Ratio) and the secondary dendrite arm spacing. Analyzing the solidification microstructure of billets plays a crucial role in optimizing process parameters and enhancing the performance of castings. Although various methods, such as dendrite corrosion, electron probes, and synchrotron radiation in situ observations [1,2,3], are available for detecting microstructures, their application in large-scale production is restricted due to their high costs and environmental constraints.

In recent decades, several numerical simulation methods have emerged for predicting the solidification microstructures of castings, including CA [4,5,6,7], PF (Phase Field) [8,9,10], and MC (Monte Carlo) [11,12,13], etc. Of these methods, CA and PF are the most widely adopted. The PF model is primarily used for simulating the local dendrite morphology of a casting due to its requirement of a large grid size. The CA model is known for its simplicity, ease of implementation, and high calculation efficiency, making it a popular choice for simulating the microstructures of continuous casting billets. However, the solidification structures [14,15,16] simulated using the CA method have a significant drawback, i.e., dendrites grow exclusively in the direction perpendicular to the solid wall, which contradicts the actual continuous casting process where dendrites grow in different orientations. This limitation stems from mesh anisotropy, which is caused by the fixed grid layout and capture rule. Various methods have been developed to address this issue, including the random zigzag capture rule [17], block cells [18], and DCSA [19,20]. However, the first two methods are limited by the fact that they can only simulate dendrites with the same orientation simultaneously. The CA model coupled with DCSA is effective for simulating dendrites with different orientations simultaneously by dynamically tracking the dendrite tip, and has been successfully used to simulate the growth of columnar and equiaxed dendrites [21,22,23]. However, existing studies have focused on simulating the growth of single or multiple dendrites, with no research yet on simulating the microstructure of continuous casting billets. The scarcity of research in this area stems from DCSA’s requirement of a precise grid size and the computational complexity introduced by dynamic changes in capture positions and conditions. Consequently, simulating the microstructure of continuous casting billets with this approach demands immense computational power, rendering it a time-consuming endeavor or altogether unfeasible on a CPU. Therefore, improving the calculation of efficiency is the key to establishing a CA model with a low grid anisotropy capable of simulating the microstructure of continuous casting billets.

In related research fields, several methods have been proposed to improve the calculation efficiency, including parallel computing with multi-core CPUs [24,25,26,27] and adaptive mesh methods [28,29]. However, these methods are based on CPUs, the speed of which is not ideal because the number of cores is limited by the hardware architecture and they incur significant overheads when launching multi-threaded processes [30]. Recently, GPUs have become increasingly popular for accelerating large-scale calculations, owing to their multiple-core architecture with a particularly high number of lightweight cores [31,32,33,34,35]. Moreover, the CA model is particularly suitable for parallel computations using a GPU because of its discrete time and space, and the homogeneous and asynchronous application of transition rules among cells [36]. The use of heterogeneous GPU–CA models has enhanced the performance of computational models in various fields [37,38,39,40].

To effectively address the challenges posed by grid anisotropy and computational efficiency in simulating the microstructure of continuous casting billets, this paper proposes a GPU-accelerated CA-DCSA method based on our previous GPU acceleration scheme [16]. In addition to porting the serial program to the heterogeneous architecture with a refactored parallel algorithm, we further optimized the memory access efficiency by changing the memory access mode and minimizing warp divergence. Compared to previous models, the present work can simulate more detailed microstructures with clear dendrite morphologies. Moreover, the simulation results agree well with industrial experimental data in both structure (ECR and the average secondary arm spacing) and temperature distribution. In addition, this accelerated model achieves a considerable increase in speed of 1430× compared to the serial CPU implementation, making it an invaluable tool for optimizing process parameters.

## 2. Model Description for Solidification in Continuous Casting

**Figure 1:**
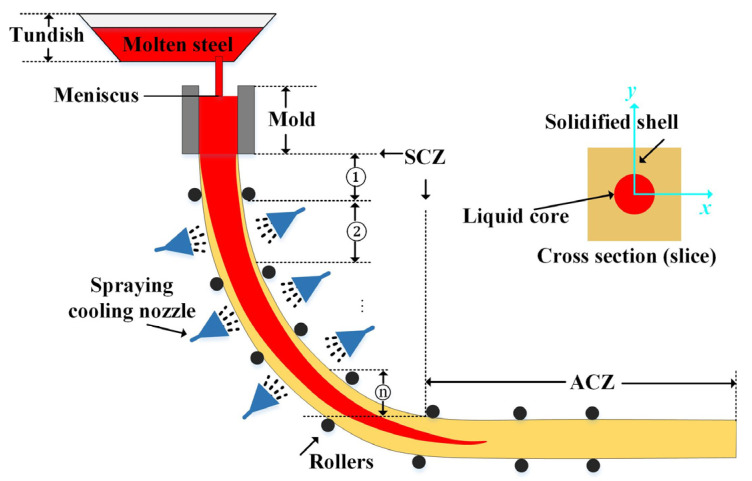
Continuous casting process.

Figure 1 illustrates the continuous casting process, wherein the molten steel flows from the tundish to the water-cooled copper mold to form the initial shell.

Then, the solidifying shell moves down to the SCZ (Secondary Cooling Zone), where it is cooled by sprayed water or an air mist. Finally, the solid strand enters the ACZ (Air-Cooling Zone) and completes the solidification process gradually. This process involves complex multi-physics coupling, including heat transfer, mass transfer, and phase change, which results in the final solidification microstructure of the continuous casting billet. This paper utilizes the “two-dimensional slice tracking” method to trace the evolution of the microstructure from the meniscus to the end of the withdrawal straightener, where the FVM (Finite Volume Method) is utilized to solve the heat and mass transfer equations, and the CA method is used to describe the phase transition process.

### 2.1. Heat and Mass Transfer Model

The heat transfer process of continuous casting billets can be described by the heat conduction equation [18,41,42]

(1) $$\rho c_{p}\frac{\partial T}{\partial t}=\frac{\partial }{\partial x}\left(\lambda \frac{\partial T}{\partial x}\right)+\frac{\partial }{\partial y}\left(\lambda \frac{\partial T}{\partial y}\right)+q$$

where T is temperature, °C; t is time, s; ρ is mass density, kg/m^3^; $c_{p}$ is equivalent specific heat; and λ is thermal conductivity. The Fe-C pseudo-binary phase diagram [43] is adopted to calculate the thermophysical properties. q is the gradient heat flux determined by different boundary conditions which are set as follows:

In the mold-cooling zone, the gradient heat flux boundary condition is given by the modified Savage–Pritchard equation [41,44].

(2) $$-\lambda \frac{\partial T}{\partial n}=A-B\sqrt{t}$$

where *A* and *B* are coefficients that can be adjusted according to the industrial data related to heat transfer conditions (heat flux, surface temperature, cooling water flow rate, and other relevant parameters that influence the heat transfer efficiency), **n** represents the normal direction, which is perpendicular to the boundary surface.

In the SCZ, heat is taken away from the billet surface by cooling water and thermal radiation, where the boundary condition can be determined by [34,41]

(3) $$-\lambda \frac{\partial T}{\partial n}=h_{i}\left(T-T_{w}\right)+\varepsilon \sigma \left(T^{4}-T_{a}^{4}\right)$$

where $h_{i}$ is the comprehensive heat transfer coefficient, the subscript i denotes the i th section in SCZ, ε is the emissivity of strand surface, with a value of 0.8; σ is the Stepan-Boltzmann constant, equal to 5.67 × 10^−8^ (W/m^2^)K^4^; $T_{w}$ and $T_{a}$ are cooling water temperature and ambient temperature, respectively, both equal to 25 °C. There are two kinds of cooling in the SCZ, i.e., water sprays and air-mist sprays, for which the coefficient $h_{i}$ is defined by Equation (4), describing two conditions [34,41].

(4) $$\left\{\begin{array}{l} h_{i(water)}=\frac{1570w_{i}^{0.55}\left[1-0.0075\left(T_{w}-273\right)\right]}{\alpha_{i}}\\ h_{i(air-mist)}=\frac{1000w_{i}}{\alpha_{i}} \end{array}\right.$$

where $w_{i}$ is the water flow density, and $\alpha_{i}$ is the machine-dependent calibration parameters that can be adjusted to minimize the difference between the simulated and industrial data. The specific application of air-mist cooling and water cooling needs to be determined according to the design of the continuous casting machine and the process’ requirements.

In the ACZ, heat is extracted through thermal radiation [34,41,42]

(5) $$-\lambda \frac{\partial T}{\partial n}=\varepsilon \sigma \left(T^{4}-T_{a}^{4}\right)$$

The solute diffusion is governed by [14,23]

(6) $$\frac{\partial C_{i}}{\partial t}=\frac{\partial }{\partial x}\left(D_{i}\frac{\partial C_{i}}{\partial x}\right)+\frac{\partial }{\partial y}\left(D_{i}\frac{\partial C_{i}}{\partial y}\right)+C_{i}\left(1-k\right)\frac{\partial f_{s}}{\partial t}$$

where $D_{i}$ means the solute diffusion coefficient calculated by Equation (7), and $C_{i}$ is the concentration, the subscript i represents two states, i.e., l is liquid and s is solid. The gradient solute flux boundary condition is 0.

(7) $$D_{i}=\left\{\begin{array}{ll} 0 & ,\;f_{s}=1\;\\ f_{s}D_{s}+(1-f_{s})D_{l} & ,\;f_{s}<1 \end{array}\right.$$

### 2.2. Phase Transition Model

In the CA method, the calculation domain is uniformly divided into square cells. The nucleation density of a cell with respect to the melt undercooling ΔT is given by Gaussian function [14,23]:

(8) $$\frac{dn}{d(\Delta T)}=\frac{n_{\max}}{\sqrt{2\pi }}\exp{\left[-\frac{1}{2}\left(\frac{\Delta T-\Delta T_{N}}{\Delta T_{\sigma }}\right)\right]}^{2}$$

where $\Delta T_{N}$ and $\Delta T_{\sigma }$ are the average and standard deviation nucleation undercooling, and $n_{\max}$ is the maximum nucleation density. The nucleation probability of a liquid cell is calculated by:

(9) $$P_{n}=\int_{\Delta T^{n}}^{{\Delta T}^{n+1}}\frac{\mathrm{dn}}{d\left(\Delta T^{\prime }\right)}d\left(\Delta T^{\prime }\right)\Delta x$$

where Δx is cell size.

To reduce the mesh anisotropy, the current CA model utilizes the DCSA rule to capture neighbors. If the $P_{n}$ of the cell labeled as “A” exceeds a randomly generated number (0~1), a nucleation event in cell “A” will be triggered. A square with a random orientation θ[−45°,45°] is subsequently placed in the center of cell “A”, and its state will be updated from liquid to interface. Then, the square will grow along the orientation θ with the velocity given by the KGT model [15]:

(10) $$v=\alpha_{1}\Delta T^{2}+\alpha_{2}\Delta T^{3}$$

where $\alpha_{1}$ and $\alpha_{2}$ are growth coefficients [45]. The melt undercooling ΔT consists of thermal, solute, and curvature undercooling, which is calculated by [18,22,23]:

(11) $$\Delta T=T_{l}-T+m\left(C_{l}-C\right)-\Gamma Kf\left(\varphi ,\theta \right)$$

(12) $$K=\frac{\left(2\partial_{x}f_{s}\partial_{y}f_{s}\partial_{xy}f_{s}-\partial_{x}{f_{s}}^{2}\partial_{yy}f_{s}-\partial_{y}{f_{s}}^{2}\partial_{xx}f_{s}\right)}{{\left(\partial_{x}{f_{s}}^{2}+\partial_{y}{f_{s}}^{2}\right)}^{3/2}}$$

(13) $$f\left(\varphi ,\theta \right)=1-15\varepsilon \cos\left(4\left(\theta -\varphi \right)\right)$$

(14) $$\varphi =\left\{\begin{array}{ll} \cos^{-1}\left({\partial_{x}f_{s}/\left(\partial_{x}{f_{s}}^{2}+\partial_{y}{f_{s}}^{2}\right)}^{1/2}\right) & ,\partial_{x}f\geq 0\\ 2\pi -\cos^{-1}\left({-\partial_{x}f_{s}/\left(\partial_{x}{f_{s}}^{2}+\partial_{y}{f_{s}}^{2}\right)}^{1/2}\right) & ,\partial_{x}f<0 \end{array}\right.$$

where $C_{l}$ and C are actual and initial concentration, wt.%; T and $T_{l}$ are the melt and liquidus temperatures, and m is the liquidus slope; Γ is the Gibbs–Thomson coefficient; K is the interface curvature; $f\left(\varphi ,\theta \right)$ is the interface anisotropy function; φ is the interface normal angle; and θ is the preferential growth orientation.

As cell “A” grows, its corners penetrate the neighboring cells and convert them into interface cells, with new squares generated at the capturing points. The square, located in cell “A” will keep expanding until its solid fraction *f_s_* reaches 1 and the state of cell “A” will change to solid. The nucleation, growth, and capture processes will continue until solidification is complete.

### 2.3. Analysis of Computational Intense

To accurately model the capture process, the DCSA requires a fine grid division. For the billet properties that possess four-fold symmetry, this paper performs dynamic simulation on a quarter section (80 mm × 80 mm) in the top right corner of the billet. In the case of a casting speed of 1.75 m/min, a grid-independent solution can be obtained when the slice is divided into 8000 × 8000 cells with the size of 10 μm. And, the time step *dt* is set to 0.0005 s to keep the calculation accuracy. As the casting process is 16 m long, there are approximately 1.1 × 10^6^ slices equating to 7.04 × 10^13^ cells. Furthermore, the dynamic monitoring of the four corners of each square within the interface cells during the intricate three-physics coupling reaction necessitates a substantial increase in computational speed. In a test of 1000 slices on Intel^®^ Xeon^®^ CPU E5-2680 v4 @2.4 GHz, the simulation process took 10.06 h. In this scenario, the whole simulation process would take about 485 days, which is unacceptable and infeasible. Therefore, improving the calculation efficiency is crucial to simulate the microstructure of continuous casting billets with a multi-orientation dendrite morphology.

## 3. GPU-Accelerated Method for CA-DCSA Model

Here, the GPU-accelerated method consists of two main components: parallelization and optimization. We begin with parallelizing the serial program using the reengineered CA-DCSA algorithm and porting it to a heterogeneous parallel architecture. Then, a series of optimization techniques is devised to enhance memory utilization and prevent divergence, thereby facilitating the effective utilization of GPU resources and enabling high-performance computations.

### 3.1. Parallelization with Redesigned Algorithm

#### 3.1.1. Porting to the Heterogenous Architecture

**Figure 2:**
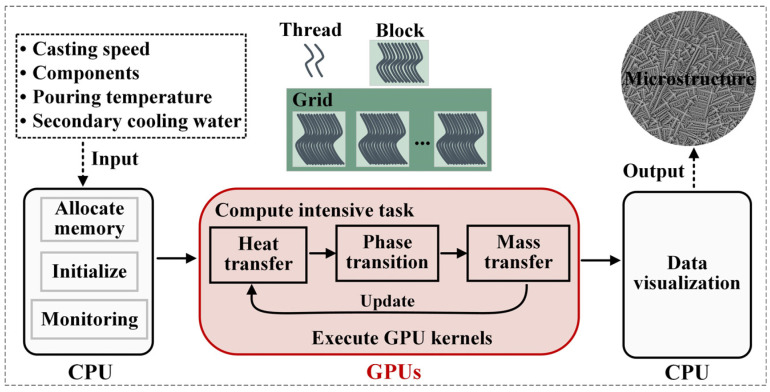
Heterogenous programming framework.

The heterogeneous model is implemented on Compute Unified Device Architecture (CUDA), a general-purpose parallel programming model consisting of a CPU code and a GPU code. First, taking full advantage of the computational power of the GPU provided by thousands of processing cores, our implementation strategically assigns the computation-intensive tasks to the GPUs, while allocating memory, initialization, program flow monitoring, and post-processing to the CPU, as shown in Figure 2. The GPU functions as a coprocessor and processes tasks through a parallel thread execution of a set of instructions referred to as a kernel. CUDA has a two-level thread hierarchy decomposed into thread blocks and grids of blocks. Once a kernel is launched, all independent threads in a grid will execute the instructions concurrently. Since the communication between CPU and GPU is very expensive, we designed only two communications, i.e., before and after the computation-intensive task. Moreover, a GPU-friendly SoA structure with all variables stored in a linearized form was utilized to organize the data as it provided the best-coalesced access pattern. In addition, read-only data that pertain to material properties, such as constant parameters and boundary conditions, were cached in constant memory. This type of memory is accessible from all threads within a grid, which can significantly reduce the number of memory transactions and improve overall performance. By caching these data in constant memory, they can be quickly accessed by multiple threads without repeated memory transfers from the global memory, which can lead to a significant reduction in memory latency. After the aforementioned parallelization operation, the program can be ported to the heterogenous architecture.

The functions running on a GPU consist of three main parts, as shown in Figure 2, and each part is implemented by several kernel functions; the solution is shown in the following pseudocode:



$\begin{array}{l} \mathrm{Compute}\;\mathrm{intensive}\;\mathrm{task}\\ 1.\mathrm{Heat}\;\mathrm{transfer}\;(\mathrm{Input}\text{:}\;f_{s})\\ getb\text{:}\;\mathrm{boundary}\;\mathrm{conditions}\\ ThermaPro\text{:}\;\mathrm{thermal}\;\mathrm{properties}\\ getT\text{:}\;\mathrm{temperature}\;\mathrm{for}\;\mathrm{Finite}\;\mathrm{Volume}\;\\ 2.\;\mathrm{Phase}\;\mathrm{transition}\;(\mathrm{Input}\text{:}\;T,\;C_{l})\\ getmT\text{:}\;\mathrm{temperature}\;\mathrm{for}\;\mathrm{cell}\\ calCur\text{:}\;\mathrm{curvature}\\ caldT\text{:}\;\mathrm{undercooling}\\ nucleation\text{:}\;\mathrm{nucleation}\;\mathrm{probability}\\ soliden\text{:}\;\mathrm{capture}\;\mathrm{process}\\ growth\text{:}\;\mathrm{growth}\;\mathrm{velocity}\\ 3.\;\mathrm{Mass}\;\mathrm{transfer}\;(\mathrm{Input}\text{:}\;T,\;f_{s})\\ soluteRedi\text{:}\;\;\mathrm{solute}\;\mathrm{redistribution}\;\mathrm{in}\;\mathrm{the}\;S/L\;\mathrm{interface}\\ soluteAdd\text{:}\;\mathrm{load}\;\mathrm{solute}\;\mathrm{discharged}\;\mathrm{by}\;\mathrm{neighbors}\\ soluteDif\text{:}\;\;\mathrm{solute}\;\mathrm{diffusion}\;\mathrm{in}\;\mathrm{the}\;\mathrm{whole}\;\mathrm{domain}\\ 4.\;\mathrm{Updata} \end{array}$



This process will be repeated until the solidification is completed.

#### 3.1.2. Redesigning the Capture Process

**Figure 3:**
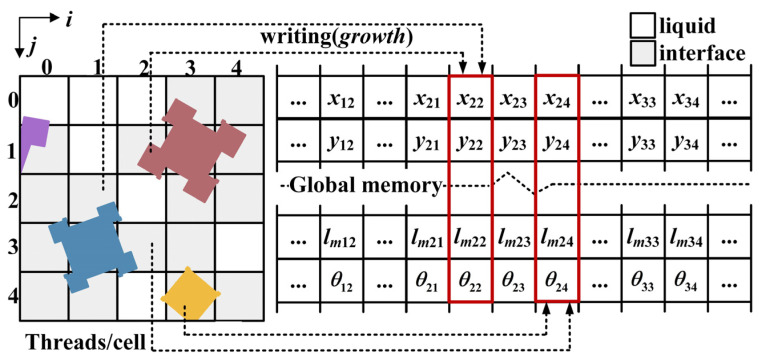
Data competition in DCSA ((*x_ij_*, *y_ij_*): the nucleation position, *l_mij_*: the maximum half diagonal length, *θ_ij_*: preferential orientation, different color represents different orientation).

The simplest way to achieve a high computational efficiency is assigning each thread to a computational unit, built on the foundation of high parallelism. To achieve this, the explicit formulation is utilized for the heat and mass transfer equations. In addition, the data competition that exists in the DCSA capture process when there are multiple neighboring cells that satisfy the capture condition can lead to undefined errors if it cannot be handled reasonably, rendering acceleration ineffective. As shown in Figure 3, at a certain time, cells (3, 4) and (2, 3) have penetrated into (2, 4), and cells (2, 1) and (1, 2) have touched (2, 2). In this case, the threads mapping to cells (3, 4) and (2, 3) will write data onto the memory space of cell (2, 4), and the threads mapping to cell (1, 2) and (2, 1) will write data onto the memory space of cell (2, 2) simultaneously. The *AtomicExch* function provided by CUDA can effectively prevent race conditions among multiple threads, but it comes with the cost of sacrificing computational parallelism and efficiency.

**Figure 4:**
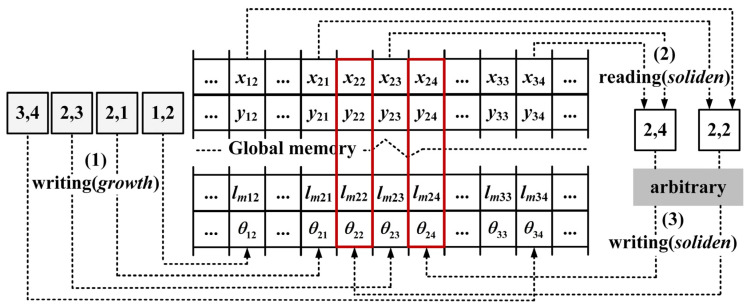
Scheme for the new parallel CA-DCSA algorithm.

To eliminate data competition without sacrificing computational parallelism, we redesigned the capture process. As shown in Figure 4, the new parallel CA-DCSA algorithm focuses on preventing threads from modifying data that belong to other threads by dividing the capture process into three primary steps:

(1) *Growth* kernel: Interface cells grow and update their growth-related parameters in global memory. No neighbor capture occurs at this stage, even if the capture condition is met.
(2) *Soliden* kernel: Liquid cells read the growth-related parameters from neighboring interface cells, and judge which neighbor satisfies the capture condition based on them. An arbitration mechanism resolves conflicts when multiple neighbors satisfy the capture condition for the same liquid cell. Also, it is crucial to determine which corner touches the current cell because different corners have different capture positions, as shown in Figure 3 where both corners of the square in cell (0, 1) can touch cell (0, 0).
(3) *Soliden* kernel: Liquid cells update their growth-related parameters based on the arbitration results from *the soliden* kernel.

In this new algorithm, threads only modify their own data, which can fundamentally avoid data competition without affecting computational parallelism.

### 3.2. Optimizations

Preliminary analysis shows that the present model suffers from a low memory access efficiency caused by a large number of memory transactions and warp divergence. Thus, to address these two issues, optimizations are performed to maximize GPU utilization and achieve effective acceleration.

#### 3.2.1. Managing Memory Accessing

Memory write operations typically incur a higher latency than read operations. This occurs because write operations require data transfer from processors/registers to memory, while read operations can directly fetch data from memory. In our implementation, a lot of variables need to be updated in the kernel “*Update*” after each time step, rendering it the most time-consuming part. Most of the updated variables are assigned by the solute redistribution process, which sets nine intermediate variables ${dC}_{i}\left(i=0,1,\ldots ,8\right)$ for each cell to store the amounts of excess solute being distributed to neighboring cells and the current cell, as shown in the naive algorithm of Algorithm 1 where *dC* is the total rejected solute by the current cell. After being used in the kernel “*SoluteAdd*”, these intermediate variables must be reset to zero to use the next slice, leading to a large number of memory writing transactions. To avoid writing memory frequently, the operation in *soluteAdd* is redesigned, which is noted as the redesigned version in Algorithm 1.

**Algorithm 1.** Two methods for solute redistribution at S/L interface.

$\begin{array}{l} naive\;version\\ \;\;\;soluteAdd\\ \;\;\;1.C_{l}+=\sum_{i=0}^{8}d{C^{i}}_{idxi}/(1-f_{s\;idx});\\ \;\;\;Update\\ \;\;\;\;2.\;dC^{i}=0;\\ \;\;\;\;3.\;dC=0;\\ red\;esigned\;version\\ \;\;\;soluteAdd\\ \;\;\;1.C_{l}+=\sum_{i=0}^{8}d{C^{i}}_{idxi}(dC_{idxi}>0)/(1-f_{s\;idx});\\ \;\;\;Update\\ \;\;\;2.\;dC=0; \end{array}$



In this new algorithm, we implement a logical expression $dC_{idxi}>0$: when the expression is evaluated to be true, it returns 1 (preserving the current time step’s value); otherwise, it returns 0 (forcing the value to zero). Therefore, the memory writing of ${dC}_{i}\left(i=0,1,\ldots ,8\right)$ is replaced by memory reading of dC. Furthermore, we optimize data reuse through instruction reordering, significantly improving memory access efficiency.

#### 3.2.2. Avoiding Warp Divergency

GPUs organize threads into warps, where each warp comprises 32 threads executing in lockstep via an SIMT (Single Instruction Multiple Thread) architecture. However, warp divergence occurs when threads within a warp take different execution paths during conditional operations, severely degrading performance [46]. In this work, conditional operations occur when solving Equations (7) and (14) using an if statement in naive version of Algorithm 2. Thus, in order to remove if conditions, we combine conditions into computational statements in the redesigned version of Algorithm 2.

**Algorithm 2.** Two methods for solute diffusion coefficient and interface normal angle.

$\begin{array}{l} naive\;version\\ \mathrm{coeiffocient}\;\mathrm{of}\;\mathrm{solute}\;\mathrm{diffusion}\;\\ \;\;\;1.\;if(f_{s}==1)\;then\\ \;\;\;2.\;\;\;\;D=0;\\ \;\;\;3.\;else\\ \;\;\;4.\;\;\;\;D=f_{s}D_{s}+(1-f_{s})D_{l};\\ \;\;\;5.\;endif\\ \mathrm{interface}\;\mathrm{normal}\;\mathrm{angle}\\ \;\;\;6.\;if\;(\partial_{x}f_{s}\text{>}0)\;then\\ \;\;\;7.\;\;\mathrm{angle}=\mathrm{acos}(\partial_{x}f_{s}/\;\mathrm{sqrt}(\partial_{x}{f_{s}}^{2}+\partial_{y}{f_{s}}^{2}))\;\\ \;\;\;8.\;elseif\;(\partial_{x}f_{s}||\partial_{y}f_{s})\\ \;\;\;9.\;\;\mathrm{angle}=2\pi -\mathrm{acos}(-\partial_{x}f_{s}/\;\mathrm{sqrt}(\partial_{x}{f_{s}}^{2}+\partial_{y}{f_{s}}^{2}))\\ \;\;\;10.endif \end{array}\;\begin{array}{l} red\;esigned\;version\\ \mathrm{coeiffocient}\;\mathrm{of}\;\mathrm{solute}\;\mathrm{diffusion}\;\\ \;\;\;D=(f_{s}==1)\cdot 0+(f_{s}\text{<}\;1)\cdot (f_{s}D_{s}+(1-f_{s})D_{l});\\ \mathrm{interface}\;\mathrm{normal}\;\mathrm{angle}\\ \;\;\;\mathrm{angle}=(\partial_{x}f_{s}\text{>}0)\cdot \mathrm{acos}(\partial_{x}f_{s}/\;\mathrm{sqrt}(\partial_{x}{f_{s}}^{2}+\partial_{y}{f_{s}}^{2}))+(\partial_{x}f_{s}\geq 0\\ \;\;\;\text{\&\&}(\partial_{x}f_{s}||\partial_{y}f_{s}))\cdot (2\pi -\mathrm{acos}(-\partial_{x}f_{s}/\;\mathrm{sqrt}(\partial_{x}{f_{s}}^{2}+\partial_{y}{f_{s}}^{2}))) \end{array}$



## 4. Results and Discussion

The GPU-accelerated CA-DCSA method was applied to simulate the microstructure of continuous casting billets from a certain steel plant. The simulation accuracy was validated against the industrial measurement data, including the ECR, the average secondary arm spacing, and the temperature. The relevant caster parameters and billet properties are listed in Table 1, while the chemical compositions of the reference steels are detailed in Table 2. In our case, the SCZ of the continuous caster is divided into four sections. The first two sections use water cooling, while the last two sections employ air-mist cooling.

### 4.1. Validation with Industrial Experiment

**Figure 5:**
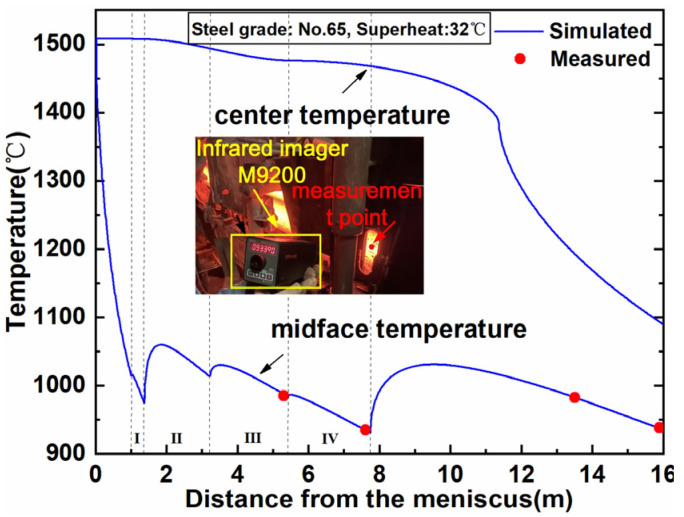
Temperature distribution during continuous casting (I: SCZ 1, II: SCZ 2, III: SCZ 3, IV: SCZ 4).

To validate model accuracy, we simulated billets 65# and 60# using a time step of 5 × 10^−4^ s and a grid resolution of 10^−5^ m. Given the critical relationship between microstructure and thermal history, temperature validation was prioritized. In order to validate the calculated temperature, a series of temperatures for steel 65# (superheat: 32 °C), were measured using an M9200 infrared imager. The temperature variation in the billet during the continuous casting process is shown in Figure 5, where the measurements are taken at the center of the arc surface. The predicted temperatures at the center of the inner arc surface show a significant reheating phenomenon in the transition cooling zone, which is caused by the differences in the boundary heat flux density due to different boundary conditions. The measured temperatures at 5.3, 7.6, 13.5, and 15.9 m away from the meniscus are 985 °C, 935 °C, 973 °C, and 941 °C, respectively, and the corresponding simulation results are 986.8 °C, 933.9 °C, 974.1 °C, and 940.2 °C. Thus, the calculated temperatures agree well with the measured ones with a maximum deviation of 1.8 °C, which is negligible compared to the temperature of the billet.

**Figure 6:**
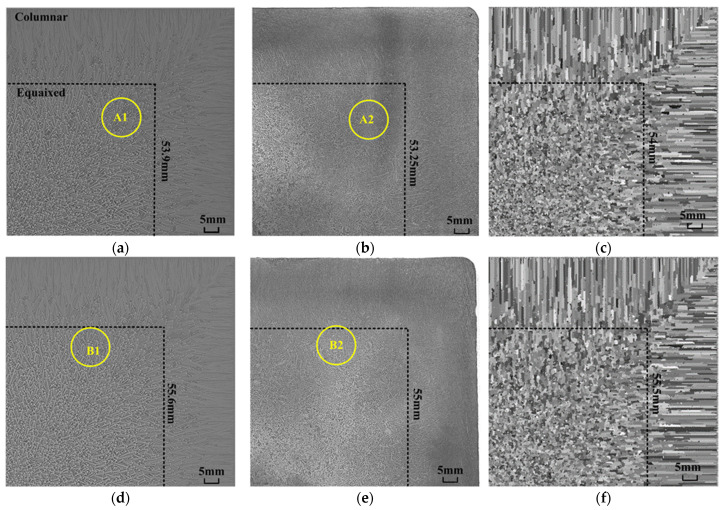
Microstructures of continuous casting billets ((**a**) simulated results for billet 65#, (**b**) industrial experiment for 65#, (**c**) simulated results of billet 65# using previous method [16], (**d**) simulated results for billet 60#, (**e**) industrial experiment for 60#, and (**f**) simulated results for billet 60# billet using previous method [16]).

Moreover, the microstructures of continuous casting billets 65# and 60# were simulated and experimentally validated. To evaluate the method’s effectiveness, industrial billet specimens were etched using a picric acid–hydrochloric acid solution, producing low-magnification micrographs (Figure 6b,e). Also, the simulated structures of continuous casting billets from our previous work are displayed as Figure 6c,f, where all dendrites grow perpendicular to the solid wall and we hardly observed a dendrite morphology. Here, the present model can simulate a clear dendrite morphology with not only multi-orientation dendrites but also a secondary dendrite arm, as shown in Figure 6a,d, showing excellent agreement with experimental observations.

To quantitatively analyze the accuracy of this method, we first calculated the ECR at the macroscopic level and the average secondary arm spacing within a selected region at the microscopic level. The structure of the billets has a typical columnar crystal zone and an equiaxed crystal zone, which are distinguished by the average ratio *α* of the short axis to the long axis [47]. The ECR are calculated using

$$\frac{\mathrm{area}\;\mathrm{of}\;\mathrm{the}\;\mathrm{equixed}\;\mathrm{crystal}\;\mathrm{zone}}{\mathrm{total}\;\mathrm{area}}\times 100\%$$

The ECRs of the microstructure are listed in Table 3, where ECRS is the simulated result, ECRE represents the experimental result, and RE is the relative error between them. It can be concluded that the simulated and experimental ECRs match well with the relative errors of 2.5% and 2.1%.

Moreover, to enhance the observation of microscopic dendrites, circular regions (radius = 6.46 mm) were selected and enlarged in Figure 6. Regions A1 and A2 are positioned 41mm from the upper boundary and 38.5 mm from the left boundary, B1 and B2 are located at a distance of 30.5 mm from the upper boundary and 30 mm from the left boundary, as depicted in Figure 6. It is clear that the dendrite morphologies in the simulation results are not exactly the same as the dendrites in the industry experiment, and these differences arise from natural randomness in both nucleation processes. Therefore, our analysis adopts a statistical comparison approach to compare the simulation result to the experiment result.

The experimental dendrite morphology exhibits a lower clarity compared to the simulations, and there are two main reasons for this. Two primary factors contribute to this: (1) experimental artifacts including localized corrosion defects; (2) image acquisition artifacts introduced during optical microscopy. To improve the clarity of the results, negative film processing and other adjustments were applied to the experimental images.

**Figure 7:**
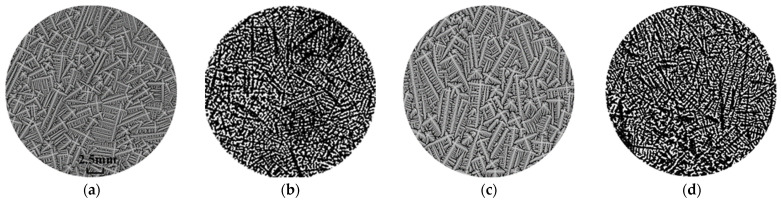
Zoomed-in image of Figure 6 ((**a**) A1, (**b**) A2, (**c**) B1, (**d**) B2).

Comparisons will be conducted from the dendrite distribution and the average secondary arm spacing. Firstly, in the actual casting process, as shown in Figure 7b,d, dendrites are observed to be randomly distributed, lacking any discernible trend or directionality. This randomness can be attributed to the significant variability in nucleation positions and growth orientations. The simulated results, i.e., Figure 7a,c, exhibit a similar pattern of dendrite distribution, which matches well with the experiment result. Secondly, the secondary dendrite arm spacings are measured. For the 65# casting billet, the secondary arm spacing of the simulation result ranges from 273.9 μm to 314 μm with an average value of 291.6 μm, while the experiment result ranges from 280.2 μm to 321.4 μm with the average of 298.6 μm. For the 60# casting billet, the secondary arm spacing of simulation result ranges from 217.1 μm to 228 μm with the average value of 223.4 μm, while the experiment result ranges from 216.2 μm to 225.5 μm with an average of 221.8 μm. The relative errors in the average secondary arm spacing between the simulated and experimental values were 2.3% (65#) and 0.7% (60#). These results demonstrate that the proposed model can reproduce the microstructure of continuous casting billets with detailed dendrite morphologies, which agrees well with the experimental result.

### 4.2. Performance Analysis

In this section, a series of computation experiments are performed to evaluate the effectiveness of the proposed method. It is worth noting that the block size configured when launching the kernel function has significant impact on the computational performance, since the number of threads in a block generally influences the usage of the on-chip resources. So, before comparing the runtime for any case in this section, we manually adjusted the block size to obtain the optimal configuration for each kernel.

To ensure the computational reliability of the GPU algorithms, we first carry out a comparison of the temperatures calculated between the serial CPU algorithm and the GPU algorithms. Here, we have demonstrated that both GPU algorithms have the same calculation accuracy. Figure 7 depicts the temperature deviations at the center and midface of the billet throughout the continuous casting process, where the maximum difference is 3 × 10^−4^ °C, which can be considered negligible in comparison to the billet’s temperature. So, the temperatures calculated using the serial CPU algorithms and GPU algorithms can be considered to be equivalent.

**Figure 8:**
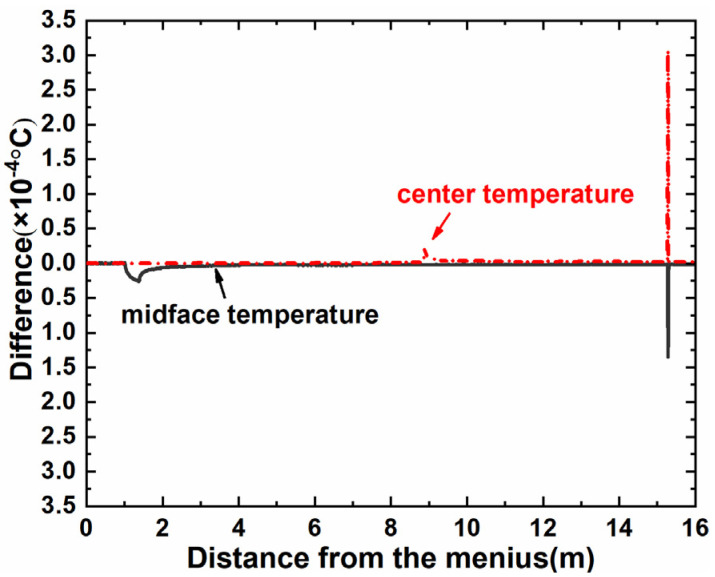
Temperature difference between serial CPU and GPU algorithms.

Then, simulations with 1000 time steps on a different number of grids were executed with three different methods, i.e., the serial CPU algorithm, basic GPU algorithm, and optimized GPU algorithm. Note that the serial CPU algorithm is run on the Intel^®^ Xeon^®^ E5-2680v4 and GPU algorithms are implemented on the Tesla P100. Here, we chose the serial CPU algorithm as the baseline, and define the speedup as the ratio of the runtime on the baseline system to the runtime on the GPU system. In this section, the speedup is utilized to evaluate the computational performance of the GPU algorithms. Figure 8 shows the computational performance, including runtime and speedup. Here, CPU-serial, GPU, and GPU* represent the runtime of the serial CPU serial algorithm, basic GPU algorithm, and optimized GPU algorithm, respectively. Meanwhile, S and S* are the speedup of the basic GPU algorithm and optimized GPU algorithm.

**Figure 9:**
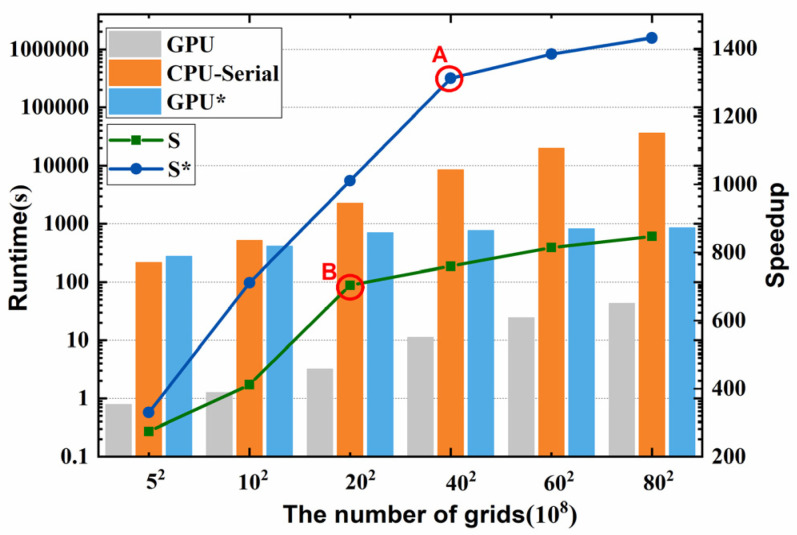
Calculation efficiency of serial CPU and GPU algorithms.

As demonstrated in Figure 9, the runtime increases with the increase in the number of grids in all three algorithms. When the number of grids increases from 5^2^ ten million to 80^2^ ten million, the runtime of the serial CPU algorithm increases steeply from 214 s to 36,229 s, while the runtime of the basic GPU algorithm increases from 0.78 s to 42.74 s, and that of the optimized GPU algorithm increases from 0.55 s to 25.32 s. Moreover, the speedup of both GPU algorithms increases with the increase in the computation domain, and the speedup increases rapidly initially, but the growth rate gradually slows down after turning points “A” and “B”. This is because, as the workload increases, the GPU can more fully utilize its parallel computing capabilities, resulting in a higher speedup. However, as the workload further increases, certain limiting factors, such as memory bandwidth and computational power limitations, may come into play, gradually restricting the growth rate of the speedup. Moreover, simply porting the serial algorithm to a heterogeneous parallel architecture always makes it impossible to fully utilize the GPU resources due to the different memory architectures between CPUs and GPUs. In the optimized GPU algorithms, we improved the memory accessing efficiency by changing the memory accessing way and avoiding warp divergency (Section 3.2), which achieved a performance improvement of about 70% with an increase in speedup from 848× to 1430×. It is fascinating to note that turning point “A” occurs later than “B”. This is because, by increasing memory utilization, the GPU can more effectively utilize its memory bandwidth, reducing the limitations imposed by memory bottlenecks on the growth of speedup. This means that the GPU can handle more data and perform more parallel computations, thereby delaying the decline in the rate of performance improvement. Therefore, by optimizing memory utilization, the occurrence of the turning point can be postponed, allowing the GPU to achieve a better speedup at higher computational workloads.

## 5. Conclusions

The proposed GPU-accelerated CA-DCSA method achieves a breakthrough performance in simulating the dendritic microstructures of continuous casting billets, with three key advancements:

(1) Computational efficiency: A 1430× speedup over serial CPU implementations enables microstructure simulations within practical timeframes, overcoming previous computational bottlenecks.
(2) Morphological accuracy: The model resolves crystal zones, dendrite orientations, and secondary dendrite arm spacing with unprecedented clarity, surpassing prior CA methods in geometric fidelity.
(3) Industrial validation: Experimental validation on steels 65# and 60# demonstrate robust agreement with measurement, with relative errors < 2.5% for equiaxed crystal ratio, secondary arm spacing, and temperature deviations less than 1.8 °C.

The successful implementation of the GPU-accelerated CA-DCSA method represents a significant advancement in microstructure simulation technology, offering valuable insights for optimizing industrial continuous casting parameters. The combination of computational efficiency and simulation accuracy makes it particularly suitable for practical applications in steel production and process development.

## Figures and Tables

**Table 1:** Parameters of caster and thermophysical properties.

Parameters	Values
Billet size (-)	160 mm × 160 mm
Steel grade (-)	65#, 60#
Casting Speed (m/min)	1.75
Effective mold length (m)	0.9
Lengths of SCZ sections (m)	0.37, 1.85, 2.20, 2.32
Liquidus temperatures (°C)	1476 (65#), 1481 (60#)
Solidus temperatures (°C)	1382 (65#), 1383 (60#)

**Table 2:** Compositions of casting steels (wt.%).

Billets	Components				
	C	Si	Mn	P	S
65#	0.65	0.24	0.59	0.22	0.08
60#	0.60	0.24	0.59	0.23	0.04

**Table 3:** The ECR of the microstructure.

65#			60#		
ECRS	ECRE	RE	ECRS	ECRE	RE
45.4%	44.3%	2.5%	48.3%	47.3%	2.1%

## Data Availability

The original contributions presented in this study are included in the article. Further inquiries can be directed to the corresponding author.
